# Fistular onion stalk extract exhibits anti-atherosclerotic effects in rats

**DOI:** 10.3892/etm.2014.1790

**Published:** 2014-06-18

**Authors:** BENHONG HE, JIANJUN HAO, WEIWEI SHENG, YUANCAI XIANG, JIEMEIA ZHANG, HAO ZHU, JINGCHENG TIAN, XU ZHU, YUNXIA FENG, HAO XIA

**Affiliations:** 1 Department of Cardiovascular Medicine, Lichuan Hospital of Traditional Chinese Medicine, Hubei University of Chinese Medicine, Lichuan, Hubei 445418, P.R. China; 2 Department of Cardiovascular Medicine, Wuhan No.1 Hospital, Huazhong University of Science and Technology, Wuhan, Hubei 430022, P.R. China; 3 Department of Cardiovascular Medicine, The Central Hospital of Enshi Prefecture, Enshi Clinical College of Wuhan University, Enshi, Hubei 445000, P.R. China; 4 Department of Cardiovascular Medicine, Renmin Hospital of Wuhan University, Wuhan, Hubei 430060, P.R. China

**Keywords:** fistular onion stalk extract, atherosclerosis, inflammatory cytokine, renin-angiotensin-aldosterone system

## Abstract

Fistular onion stalk is used as a traditional herbal medicine, and its extract exhibits certain beneficial effects on cardiovascular disease. In this study, the effects of fistular onion stalk extract on the pathological features, circulating inflammatory cytokines, local renin-angiotensin-aldosterone system (RAAS) and signaling pathway activities were examined using an *in vivo* model of atherosclerosis. Atherosclerosis of the aorta was induced by loading Sprague Dawley rats with a high-fat diet and vitamin D_2_. Fistular onion stalk extract administration began five weeks after the induction of atherosclerosis and continued for 12 weeks. Rats treated with fistular onion stalk extract showed a significant reduction in the pathological region compared with the vehicle-treated controls. Inhibition of atherosclerosis was associated with preservation of the vascular wall and immune cell infiltration. The extract also reduced the levels of the local inflammatory cytokines interleukin (IL)-1β, IL-6, monocyte chemoattractant protein-1 and tumor necrosis factor-α. Furthermore, the extract downregulated the local activity of the RAAS. In addition, extract treatment inhibited several inflammatory signaling pathways by preventing phosphorylation, including the nuclear factor κB, Janus kinase/signal transducers and activators of transcription and mitogen-activated protein kinase pathways. These data indicate that fistular onion stalk extract may be useful for the attenuation of atherosclerosis, and the mechanism includes the regulation of the local inflammatory response.

## Introduction

Atherosclerosis is a prevalent pathological process that leads to coronary and cerebrovascular diseases, two major causes of morbidity and mortality worldwide (1,2). Atherosclerosis is a progressive disorder of the walls of large and medium arteries, ranging from fatty streaks to atheromatous plaques (3). Lifestyle modifications are the first method of treatment, while medicines are usually the next step in treating atherosclerosis, among which statins are the most popular and widely prescribed (4). However, in a number of susceptible individuals the available medicine does not sufficiently prevent the progression of atherosclerosis (5,6). There is thus an urgent requirement for novel therapeutic strategies.

Abnormal lipid metabolism is a formally established, and remains the most important, risk factor for atherosclerosis (7). However, there are numerous discrepancies in the expression of the clinical disease among individuals with similar cholesterol levels (8–10). Accumulating evidence has indicated that inflammation plays a central role in atherogenesis (11–13). Macrophages were the first inflammatory cells to be associated with atherosclerosis (14). Thus far, the majority of the known leukocytes have been reported to be present in and to lead to atherosclerotic lesions (15). Cytokines are key factors during acute and chronic inflammation (16–18). The majority of these cytokines share common methods of action. The intracellular signal transduction pathways include the nuclear factor κB (NF-κB), Janus kinase/signal transducers and activators of transcription (JAK/STAT) and mitogen-activated protein kinase (MAPK) pathways (19–21). The generation of cytokine-deficient animals has provided strong evidence that cytokines play a critical role in atherosclerosis, i.e. interleukin (IL)-1β^−/−^/apolipoprotein E^−/−^ mice showed a significant decrease in the severity of atherosclerotic lesions compared with their IL-1β-expressing counterparts (22). Consistent with these findings, cytokines such as C-reactive protein, tumor necrosis factor (TNF)-α and IL-6 have been found to be associated with cardiovascular risk (23–25).

The renin-angiotensin-aldosterone system (RAAS) also plays a significant role in atherogenesis. Angiotensin II (AngII) is a major effector of the RAAS, and evidence suggests that AngII has significant proinflammatory activity (26). Blocking the effect of AngII by angiotensin-converting enzyme inhibitors (ACEIs) or AngII receptor blockers (ARBs) exerts an anti-inflammatory effect, which modifies inflammatory molecules, including intercellular adhesion molecule-1 and vascular cell adhesion molecule-1, and prevents the development of atherosclerosis in animal models (27,28).

*Allium fistulosum* is widely cultivated in Southern China and has been used to treat a variety of diseases, including the common cold and arthritis. Fistular onion stalk is derived from *A. fistulosum*, and whether or not it modulates atherosclerosis has yet to be elucidated. The present study aimed to investigate the pharmacological effect of fistular onion stalk on a rat atherosclerosis model and to determine whether an anti-inflammatory mechanism is involved in this process.

## Materials and methods

### Preparation of fistular onion stalk extract

The fresh fistular onion stalk was purchased from from local farmers in Huangpi (Wuhan, China), and identified by staff at Tongji Medical College (Huazhong University of Science and Technology, Wuhan, China). Extracts were then prepared at Tongji Medical College as described in a previous study (29). In brief, the white-sheath section was isolated and homogenized. Following centrifugation, the supernatant was filtered, frozen at −80°C and lyophilized for 24 h. The dehydrated powder was then sent for supercritical extraction by carbon dioxide, and the extraction was carried out with a laboratory scale high-pressure extraction plant (Nantong Huaan Supercritical Extraction Co., Ltd, Nantong, China). The mass of sample in the extractor was at a pressure of 30 MPa and a temperature of 50°C, and the carbon dioxide flow rate was 250–300 l/h. The separator conditions comprised a pressure of 8 MPa and a temperature of 45°C. Extracts from the same batch were used for the entire set of experiments.

### Animals and groups

Male Sprague Dawley rats (age, 12 weeks; weight, 200–250 g) were purchased from and maintained at the Division of Experimental Animals, Tongji Medical College. The care and use of the animals was approved by the Animal Care and Use Committee of Huazhong University of Science and Technology. Thirty rats were randomly divided into three groups as follows: Vehicle treatment plus non-atherosclerosis control (control, n=10), vehicle treatment plus atherosclerosis (model, n=10) and fistular onion stalk extract treatment plus atherosclerosis (treatment, n=10).

The atherosclerosis was induced by a high-fat diet and vitamin D_2_ loading as previously described (30). The model group was fed with a high-fat diet, which contained 83.3% normal food, 8% lard, 3% cholesterin, 5% plantation white sugar, 0.2% propylthiouracil and 0.5% chleolate, for 16 weeks. Vitamin D_2_ (3×10^5^ U/kg) was injected into each rat at the beginning of the experiment. The treatment group was given the same diet, and the fistular onion stalk extract was administered by gastric perfusion (20 mg/kg) from the fifth week. The control group was fed the basal diet.

The study protocol was approved by the institutional review board of Wuhan No.1 Hospital, Huazhong University of Science and Technology.

### Histopathology examination

At the end of the 16th week, the rats were kept in a fasted state for 8 h prior to anesthesia by 10% chloral hydrate (0.3 ml/100 g). The rats were then sacrificed and the aortas were separated, removed and cut open. A section of the aortas was immediately sent for cryostat sectioning, and stained by oil red for the demonstration of lipids. The other section was fixed in 4% paraformaldehyde, embedded in paraffin, further sectioned and then mounted on glass microscope slides. Pathological changes in the aorta were determined by hematoxylin and eosin staining. Atherosclerosis was analyzed in a blinded manner using four cross-sections from each specimen at intervals of 40 μm.

### Immunohistochemistry

Paraffin-embedded aortas were made into serial 5-μm cross-sections. The sections were firstly deparaffinized and rehydrated. Antigen retrieval was then performed by heating the slides in a microwave oven in 0.1 M citrate buffer (pH 6.0) for 20 min. The endogenous peroxidase activity was blocked with 3% hydrogen peroxide for 30 min. Subsequent to incubation with 2% bovine serum albumin, the slides were incubated in a humid chamber with the respective primary antibodies, at 4°C, overnight. The following antibodies were used: Anti-AngII (H-002-12; Phoenix Pharmaceuticals, Inc., Burlingame, CA, USA) at a dilution of 1:200, anti-AngII type 1 receptor (AT1) (ADI-905-743; Enzo Life Sciences, Exeter, UK) at a dilution of 1:500 and anti-AT2 (ADI-905-746; Enzo Life Sciences) at a dilution of 1:500. The slides were then incubated for 45 min at 37°C with horseradish peroxidase-conjugated secondary antibody. Diaminobenzidine was used as the chromogen substrate and Harris hematoxylin as the counterstain. Primary antibodies were substituted with phosphate-buffered saline in the negative controls. The expression was assessed semi-quantitatively on a scale of 0–3, as follows: 0, negative; 1, weak intensity staining; 2, medium intensity staining; and 3, strong intensity staining. Evaluations were performed independently and in a blinded manner by two pathologists.

### Western blot analysis

Protein was extracted using lysis buffer and quantified using the bicinchoninic acid method. Equal amounts of protein from each sample were heated for 10 min at 95°C in sample buffer and then resolved using SDS-PAGE gels and subsequently transferred to polyvinylidene difluoride membranes by semi-dry transfer. The membranes were blocked in a 5% milk solution and incubated with primary antibody at 4°C overnight. The following antibodies were used at a dilution of 1:1,000 : Anti-phosphorylated-(p-)p65 (sc-101749), anti-p65 (sc-8008), anti-p-stat3 (sc-135649), anti-stat3 (sc-8019), anti-p-p38 (sc-7973) and anti-p38 (sc-7149) (Santa Cruz Biotechnology, Inc., Santa Cruz, CA, USA). The anti-GAPDH antibody (ab9485; Abcam, Cambridge, MA, USA) was used at a dilution of 1:2,500. The membranes were then washed and incubated with horseradish peroxidase-conjugated secondary antibody. The immunoreactivity was detected by enhanced chemiluminescence. Images were captured and quantified using National Institutes of Health (NIH) Image 1.61 software (NIH, Bethesda, MD, USA).

### Quantitative polymerase chain reaction (PCR)

The total RNA was isolated and reverse transcribed (Applied Biosystems, Inc., Foster City, CA, USA). Quantitative (q)PCR was then performed using an ABI 7900 System (Applied Biosystems, Inc.) in the presence of SYBR Green (Applied Biosystems, Inc.). The primer sequences are were follows: AngII (forward): 5′-CCGCATTTAACTGCTCACACA-3′, (reverse):5′-ATCATGTAGTAGAGAACAGGAATTGCTT-3′; AT1 (forward): 5′-GCAGCACTTCACTACCAAATGGGC-3′, (reverse) 5′-CAGGACAAAAGCAGGCTAGGGAGA-3′; AT2 (forward): 5′-GGAAGGTAGAACATACATTAAATG-3′, (reverse): 5′-AGAGAAACAGCAGCTAAAGAATT-3′. Target sequences were amplified at 95°C for 10 min, followed by 40 cycles of 95°C for 15 sec and 60°C for 1 min. GAPDH was used as an endogenous normalization control. All the assays were performed in triplicate. The fold change in mRNA expression was determined according to the 2^ΔΔCt^ method.

### ELISA

The affected aorta of the rats was obtained and sent for the measurement of cytokine levels. The quantification was determined using quantitative IL-1β, IL-6, monocyte chemoattractant protein-1 (MCP-1) and TNF-α ELISA kits (RLB00, R6000B, MJE00 and RTA00, respectively; R&D Systems, Minneapolis, MN, USA) according to the manufacturer’s instructions. The results were expressed using the optical density value of the tissue.

### Statistical analysis

All data are presented as the mean ± standard deviation. Statistical analyses were conducted using SPSS 13.0 software (SPSS, Inc., Chicago, IL, USA). The significance of differences was calculated by a one-way analysis of variance test followed by a least significant difference post hoc analysis. The level of statistical significance was set at P<0.05.

## Results

### Fistular onion stalk extract prevents the progression of atherosclerosis

**Figure 1:**
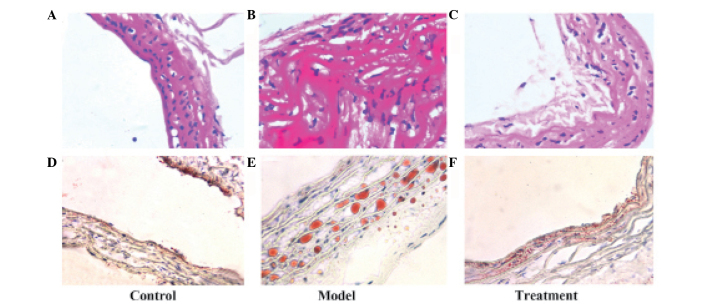
Effect of fistular onion stalk extract on atherosclerosis. (A–C) Histological examination of the rat aorta of the (A) Control, (B) Model and (C) Treatment groups by hematoxylin and eosin staining. (D–F) Examination of lipidoses in the rat aorta of the (D) Control, (E) Model and (F) Treatment groups using the oil red stain. Each section shows a representative of 10 samples with similar results.

The lesion area in the transverse section of the aorta was expressed as a percentage of the lesion to the total area of the aortic tissue. No significant atherosclerotic lesions were identified in the control group; however, the model rats exhibited significantly more extensive lesions at the thoracic and abdominal aortic regions. Evident endothelium damage, smooth muscle proliferation and lymphocyte infiltration was observed (Fig. 1). Lesion formation at both locations was significantly reduced after 12 weeks of treatment (P<0.05). The average lesion area was 1.72, 64.7 and 24.9% for the control, model and treatment groups, respectively (Fig. 1 and Table I). It was also revealed that treatment with fistular onion stalk extract significantly reduced lipidoses compared with the model group (P<0.05, Table I). Aortic cross-sections stained for lipid lesions are shown in Fig. 1.

### Fistular onion stalk extract attenuates levels of local inflammatory cytokines in aortic tissue

**Figure 2:**
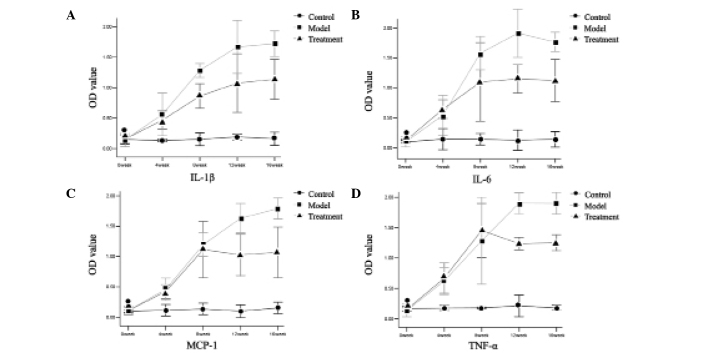
Attenuation of inflammatory cytokine levels by fistular onion stalk extract. (A–D) Dynamic changes in local (A) IL-1β, (B) IL-6, (C) MCP-1 and (D) TNF-α in aortic tissue among the different groups. IL, interleukin; MCP-1, monocyte chemoattractant protein-1; TNF-α, tumor necrosis factor-α; OD, optical density.

The differences in local cytokine levels in the aortic tissue among the three groups were determined. As shown in Fig. 2, atherosclerosis induced by a high-fat diet was characterized by a gradual increase in the levels of the inflammatory cytokines IL-1β, IL-6, MCP-1 and TNF-α compared with the control group. However, the levels of these cytokines were gradually downregulated following fistular onion stalk extract treatment, compared with the effects observed in the model group, and this difference was statistically significant at the end of the 16th week.

### Fistular onion stalk extract inhibits local RAAS activity in aortic tissue

**Figure 3:**
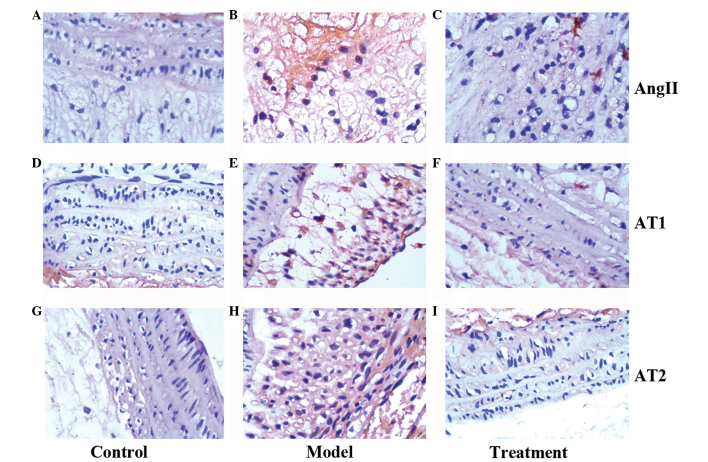
Immunohistochemical detection of the local expression of members of the renin-angiotensin-aldosterone system. (A–C) AngII, (D–F) AT1 and (G–I) AT2 expression among the different groups. AngII, angiotensin II; AT1, AngII type 1 receptor; AT2, AngII type 2 receptor.

**Figure 4:**
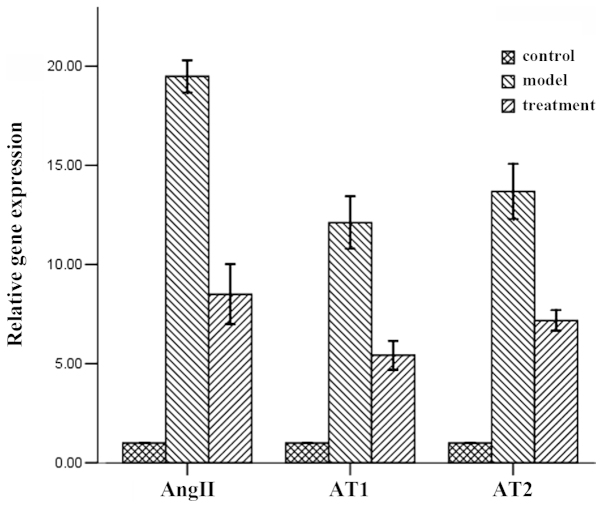
Quantitative polymerase chain reaction analysis of the local expression of members of the renin-angiotensin-aldosterone system (AngII, AT1 and AT2) among the different groups. AngII, angiotensin II; AT1, AngII type 1 receptor; AT2, AngII type 2 receptor.

To evaluate the local RAAS activity, the expression levels of AngII, AT1 and AT2 were determined by western blot analysis. The data revealed that the levels of these proteins were elevated at 16 weeks post-atherosclerosis induction; however, fistular onion stalk extract treatment resulted in a significant decrease in their expression (Fig. 3). These effects were further confirmed by a quantitative polymerase chain reaction analysis of mRNA levels (Fig. 4).

### Fistular onion stalk extract inhibits local inflammatory signaling pathways in aortic tissue

**Figure 5:**
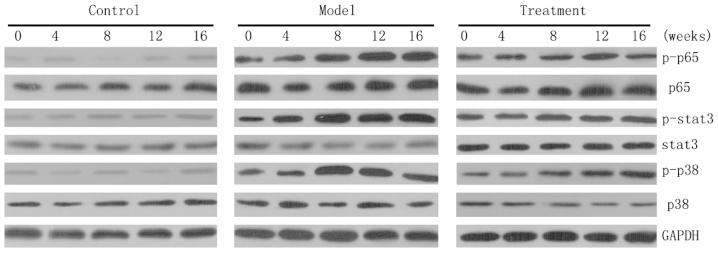
Fistular onion stalk extract inhibits local inflammatory signaling pathways. The control group maintained a low expression and the model group had a significantly upregulated expression of p-p65, p-stat3 and p-p38. However, the p-p65, p-stat3 and p-p38 expression in the treatment group was downregulated and maintained at a lower level than that in the model group. Total expression levels of p65, stat3 and p38 were similar among the different groups. GAPDH expression was set as an internal control. p-, phosphorylated-; stat3, signal transducer and activator of transcription 3.

A number of signaling pathways are believed to involve the transduction of signals from cytokines as well as the regulation of inflammatory cytokines. Three pathways were examined in the present study: The NF-κB, JAK/STAT and p38 MAPK pathways. Western blot analysis revealed that the expression levels of p-p65, p-stat3 and p-p38 were upregulated following the induction of atherosclerosis, while the total levels of p65, stat3 and p38 did not significantly differ. This indicates that these pathways are all activated through phosphorylation. The activity of these pathways was then detected in the treatment group. As expected, administration of fistular onion stalk extract was able to inhibit pathway activation (Fig. 5).

## Discussion

*A. fistulosum* L. var. *caespitosum* Makino possesses, according to the Traditional Chinese Medicine theory, the capacity for engorgement-alleviating, detoxifying and perspiration-promoting effects (31). Our previous study (32) showed that fistular onion stalk extract exhibited a potential benefit in the treatment of cardiovascular disease. In the present study, the most notable finding was that treatment with fistular onion stalk extract reduced the development of high-fat-induced experimental atherosclerosis in rats. Treatment was shown to preserve the aortic wall structure, inhibit local RAAS activity and prevent an inflammatory response. These data provide experimental evidence that treatment with fistular onion stalk extract can suppress atherosclerosis.

Continuing systemic administration of a high-fat diet appears to induce a cascade of events in the aortas, including immune cell recruitment, increased local production of cytokines and structural disruption of the aortic wall (30,33). These events lead to the recognized histological features of human atherosclerosis.

Chronic inflammation is one of the most important features involved in the development and progression of atherosclerosis. Early atherogenesis is characterized by the expression of pro-inflammatory cytokines. Cytokines such as IL-1β, IL-6, MCP-1 and TNF-α may be produced by cardiovascular cells, including endothelial and vascular smooth muscle cells, monocytes/macrophages and lymphocytes; these cytokines can, in turn, activate various cardiovascular cells (34,35). The positive feedback therefore results in a growing inflammatory response and loss of homeostasis within the vessel wall. Consistent with this, the *in vivo* model in the present study showed an enhanced local expression of IL-1β, IL-6, MCP-1 and TNF-α in the damaged vessel walls. It has been reported that the attenuation of the inflammatory response in animal models inhibits atherosclerosis (36). Therefore, the hypothesis of the present study was that fistular onion stalk extract would inhibit the expansion of atherosclerosis via a reduction in the local inflammatory response. The results demonstrated that lymphocyte infiltration was significantly reduced and local cytokines were downregulated following fistular onion stalk extract treatment.

The RAAS plays an important role in the regulation of extracellular fluid volume and sodium balance. It is now apparent that the RAAS also plays a pivotal role in the initiation and deterioration of vascular inflammatory processes, which are involved in in the formation of foam cells, the production of fatty streaks and the eventual progression to the development of rupture-vulnerable plaques (37–39). In the *in vivo* model in the present study, the expression of AngII, AT1 and AT2 was increased in the affected aorta. Antagonists of the RAAS, including ACEIs and ARBs, also exert anti-inflammatory effects (40,41); therefore, the present study investigated whether fistular onion stalk extract would influence local RAAS activity. It was revealed that the activity of AngII, AT1 and AT2 was downregulated in the treatment group compared with that in the model group.

The present study further determined the effect of fistular onion stalk extract on inflammatory signaling pathways. The NF-κB signaling pathway regulates inflammatory responses and has been implicated in atherosclerosis (42). Endothelium-restricted inhibition of NF-κB activation results in markedly reduced atherosclerotic plaque formation (43). Consistent with the above studies, the present study revealed that the p-p65 levels were elevated in the model group, but decreased following treatment with fistular onion stalk extract.

The majority of ILs, colony-stimulating factors and interferons mediate their effects through the JAK/STAT pathway. A previous study showed that the JAK/STAT pathway is an important signaling pathway regulating the initiation/progression of atherosclerosis (21), and JAK/STAT activation has been found in atherosclerotic lesions (44). In addition, when vascular cells were incubated with cytokines or AngII, the JAK/STAT pathway was activated (45,46). The present study also demonstrated that the levels of p-stat3 became elevated following atherosclerosis induction, while downregulated following fistular onion stalk extract administration.

MAPK cascades have also been reported to contribute to foam cell formation. When mouse peritoneal macrophages were treated with oxidized low-density lipoprotein (oxLDL), extracellular signal-regulated kinase 1/2, p38α MAPK and c-Jun N-terminal kinase (JNK) 1/2 were all activated within 15 min, and the treatment of macrophages with Src, JNK or p38 MAPK inhibitors blocked oxLDL-induced foam cell formation (47). The present study also found that the levels of p-p38 were increased in the atherosclerosis model group, and treatment with fistular onion stalk extract suppressed its expression.

Flavonoids are polyphenolic substances derived from plants. Considerable *in vitro* and *in vivo* animal research has focused on the anti-inflammatory potential of flavonoids, including protection against arthritis (48), acute spinal cord injury (49) and metabolic syndrome (50), as well as the anti-atherosclerotic effects of flavonoids (51). In a previous study (31), four flavonoids were successfully isolated from fistular onion stalk extract, which may explain the potent anti-inflammatory effect of fistular onion stalk extract observed in the present study. In addition, the total expression of p65, stat3 and p38 in all three groups in the present study was similar, which suggested that fistular onion stalk extract exerted its anti-inflammatory effect via the inhibition of phosphorylation.

In conclusion, the present study demonstrated that fistular onion stalk extract significantly prevents the progression of experimental atherosclerosis. This compound attenuates lymphocyte infiltration and preserves vascular structure. Furthermore, fistular onion stalk extract reduces the expression of local inflammatory cytokines and RAAS proteins and inhibits the intracellular phosphorylation of a number of key proteins in inflammatory signal transduction. Fistular onion stalk extract is thus useful as a potential therapy for atherosclerosis.

## Figures and Tables

**Table I:** Lesion areas of the different groups at the end of the experiment.

Group	Atherosclerotic area (%)	Lipidoses area (%)
Control	1.72±0.61	0.85±0.35
Model	64.7±47.48^a^,^b^	50.43±1.04^a^,^b^
Treatment	24.9±66.07	4.19±1.07

Data are presented as the mean ± standard deviation.

a P<0.05 compared with the control group;

b P<0.05 compared with the treatment group. N=10 in all three groups.
